# Human Intelligence and Polymorphisms in the DNA Methyltransferase Genes Involved in Epigenetic Marking

**DOI:** 10.1371/journal.pone.0011329

**Published:** 2010-06-25

**Authors:** Paul Haggarty, Gwen Hoad, Sarah E. Harris, John M. Starr, Helen C. Fox, Ian J. Deary, Lawrence J. Whalley

**Affiliations:** 1 Nutrition and Epigenetics Group, Rowett Institute of Nutrition and Health, University of Aberdeen, Aberdeen, United Kingdom; 2 Centre for Cognitive Ageing and Cognitive Epidemiology, Medical Genetics Section, University of Edinburgh, Edinburgh, United Kingdom; 3 Centre for Cognitive Ageing and Cognitive Epidemiology, Geriatric Medicine Unit, University of Edinburgh, Royal Victoria Hospital, Edinburgh, United Kingdom; 4 Institute of Applied Health Sciences, University of Aberdeen, Aberdeen, United Kingdom; 5 Centre for Cognitive Ageing and Cognitive Epidemiology, Department of Psychology, University of Edinburgh, Edinburgh, United Kingdom; Ludwig-Maximilians-Universität München, Germany

## Abstract

Epigenetic mechanisms have been implicated in syndromes associated with mental impairment but little is known about the role of epigenetics in determining the normal variation in human intelligence. We measured polymorphisms in four DNA methyltransferases (*DNMT1*, *DNMT3A*, *DNMT3B* and *DNMT3L*) involved in epigenetic marking and related these to childhood and adult general intelligence in a population (n = 1542) consisting of two Scottish cohorts born in 1936 and residing in Lothian (n = 1075) or Aberdeen (n = 467). All subjects had taken the same test of intelligence at age 11yrs. The Lothian cohort took the test again at age 70yrs. The minor T allele of *DNMT3L* SNP 11330C>T (rs7354779) allele was associated with a higher standardised childhood intelligence score; greatest effect in the dominant analysis but also significant in the additive model (coefficient = 1.40_additive_; 95%CI 0.22,2.59; p = 0.020 and 1.99_dominant_; 95%CI 0.55,3.43; p = 0.007). The *DNMT3L* C allele was associated with an increased risk of being below average intelligence (OR 1.25_additive_; 95%CI 1.05,1.51; p = 0.011 and OR 1.37_dominant_; 95%CI 1.11,1.68; p = 0.003), and being in the lowest 40^th^ (p_additive_ = 0.009; p_dominant_ = 0.002) and lowest 30^th^ (p_additive_ = 0.004; p_dominant_ = 0.002) centiles for intelligence. After Bonferroni correction for the number variants tested the link between *DNMT3L* 11330C>T and childhood intelligence remained significant by linear regression and centile analysis; only the additive regression model was borderline significant. Adult intelligence was similarly linked to the *DNMT3L* variant but this analysis was limited by the numbers studied and nature of the test and the association was not significant after Bonferroni correction. We believe that the role of epigenetics in the normal variation in human intelligence merits further study and that this novel finding should be tested in other cohorts.

## Introduction

Intelligence is a general mental capability which encompasses the ability to reason, plan, solve problems, think abstractly, learn quickly and make sense of our surroundings [1]. Human intelligence is characterised by a high level of heritability [1], [2] but known genetic effects can account for very little of this [1] and it has been suggested that the effect of individual genes may be much smaller than previously assumed [3]. There is growing interest in the potential for epigenetics to influence cognition [4]–[7]. Epigenetic status has recently been defined as “a stably heritable phenotype resulting from changes in a chromosome without alterations in the DNA sequence.” [8]. Such epigenetic ‘heritability’ may occur through either mitosis or meiosis and therefore has the potential to explain at least part of the high heritability of intelligence. Epigenetic mechanisms have been implicated in many syndromes associated with mental impairment; Autism (MIM209850), Rett (MIM312750), Immunodeficiency-Centromeric Instability- Facial Abnormalities (ICF) (MIM242860), Prader-Willi (MIM176270), Angelman (MIM105830), Fragile X (MIM300624), Rubinstein-Tabi (MIM180849) [4]–[6], [9]–[13] but little is known about the role of epigenetics in determining the normal variation in human cognitive abilities.

The role of epigenetics in human complex traits such as intelligence is difficult to study for a number of reasons. Epigenetic status can be influenced by factors such as diet [14] and alcohol [15] therefore, depending on the epigenetic mark of interest, there is a danger of reverse causality, where lifestyle choices linked to intelligence may influence epigenetic status. A further difficulty is that the epigenetic status of many genes is tissue specific therefore the epigenome of easily accessible tissues (e.g. blood or buccal cells) may not always reflect the epigenome of the functional organ of interest (e.g. the brain). Perhaps paradoxically, evidence for the involvement of epigenetic processes in intelligence may be obtained from genetic association studies. The goal of genetic association studies is to identify patterns of polymorphisms that vary systematically between individuals with different phenotypes and could therefore represent the effects of risk-enhancing or protective alleles [16]. Association is assumed to arise because the function of the gene has been altered in some way by the measured variant - or another in linkage disequilibrium with it - and that the change in gene function influences the phenotype. Such an approach can help to identify specific biological processes underpinning traits that may otherwise be difficult to study in humans. Analysis of genetic variants within the genes of the epigenetic pathway has been carried out to look for evidence of the involvement of epigenetic processes in the aetiology of diseases such as cancer [17]. We have used the same approach here in relation to human intelligence.

DNA methylation is probably the most commonly studied epigenetic phenomenon. We investigated the association between variants in genes involved in the epigenetic marking of DNA by methylation and childhood and adult general intelligence in population based samples with an unusually valuable phenotype: a large number of subjects who had taken the same validated general, IQ-type mental test at age 11. A sub group of these took the same test again almost 60 years later. Polymorphisms were measured in the four DNA methyltransferases: DNMT1 (MIM126375); DNMT3A (MIM602769); DNMT3B (MIM602900); DNMT3L (MIM606588). Genetic variation in all four *DNMT*s was studied in order to provide additional information on the nature of the epigenetic process which may influence human intelligence. The main function of DNMT1 is to ensure the propagation of existing methylation patterns, whilst DNMT3A and DNMT3B are primarily required for *de novo* methylation, with DNMT3L acting as an essential cofactor, particularly in the establishment of methylation imprints in the gametes [18]–[22].

## Materials and Methods

### Subjects

The Lothian and Aberdeen Birth Cohorts 1936 (LBC1936 and ABC1936) comprise surviving participants of the Scottish Mental Survey 1947 (SMS1947) who now live in the Lothian (Edinburgh and its surroundings) and Aberdeen areas of Scotland, respectively.

### Ethics statement

Ethics permission for the study was obtained from the Multi-Centre Research Ethics Committee for Scotland (MREC/01/0/56) and from Lothian Research Ethics Committee (LREC/2003/2/29) and Grampian Research Ethics Committee (LREC/01/0299). The research was conducted in compliance with the Helsinki Declaration. All subjects gave written, informed consent.

### Mental test

On June 4th 1947 almost all people born in 1936 and attending school in Scotland were tested on a valid general cognitive ability test [23]. The mental test was a version of the Moray House Test (MHT) No. 12, which was concurrently validated against the Terman-Merrill revision of the Binet Scales with a coefficient of approximately 0.8. The test was re-administered to the LBC1936 when participants were seen again at age 70 (IQR = 1.3) yrs, using the same instructions and the same 45-minute time limit. Only two small changes were made to items whose content had become archaic. The test is often referred to as the ‘Verbal Test’ or as a ‘verbal reasoning’ test. However, the test has items of a variety of types: following directions (14 items), same-opposites (11), word classification (10), analogies (8), practical items (6), reasoning (5), proverbs (4), arithmetic (4), spatial items (4), mixed sentences (3), cipher decoding (2), and other items (4). The maximum possible score in the MHT was 76.

### Variant selection and genotyping

Blood samples were taken for DNA extraction from white blood cells and the samples stored as described elsewhere [24], [25]. The concentration of DNA was determined by RNaseP assay (Applied Biosystems, Warrington, UK) before genotyping. Genotypes were detected by allelic discrimination assay using TaqMan® MGB probes labelled with 6-FAM™ and VIC® on a 7500 Fast real-time PCR system (Applied Biosystems, Warrington, UK). The gene variants studied were: *DNMT1* (MIM126375 19p13.3–p13.2; 21220C>T; rs2114724); *DNMT3A* (MIM 602769; 2p23; 28510C>T; rs734693); *DNMT3B* (MIM602900; 20q11.2; 46359C>T; rs2424913); *DNMT3L* (MIM606588; 21q22.3; 11330C>T; rs7354779). A second variant in DNMT1 (35433A>G; rs2162560) was measured in the ABC1936 cohort but this was found to be highly reciprocally correlated with the DNMT1 21220C>T variant (coefficient  =  −0.85; p<0.001) and therefore was not measured in the larger LBC1936 and dropped from the study. We used the candidate polymorphism approach which focuses on individual polymorphisms that are suspected of being implicated in biological function [16]. This hypothesis directed approach has the advantage of minimising the number of variants studied and hence reducing the risk of false positive results. Very rare variants are of limited value in association studies as they are only relevant to a small proportion of the population and their effects are difficult to detect in practice therefore we selected only variants where the frequency of the homozygous minor allele was >5%. The *DNMT3L* 11330C>T variant is a non-synonymous polymorphism resulting in the amino acid change Gly278Arg within the C-terminal portion of DNMT3L. Such non-synonymous variations in the functional domains of the DNMTs are unusual and no non-synonymous polymorphisms have been detected in the catalytic domains of *DNMT3A/B* or *DNMT1* in a European population [26]. Variants in these genes were selected for study primarily on the basis of whether they have been related to a phenotype. Such associations are assumed to arise as a result of non-coding effects [27] or because the variants are in linkage disequilibrium with functional variant(s) within the gene. The *DNMT3B* variant is located in the promoter region and has been associated with the risk of cancer [28]. For the DNMT1, 3A and 3L variants we have observed phenotypic associations in other cohorts (manuscripts in preparation). All available DNA samples from the cohorts were measured. Only samples in which a valid genotype was not able to be measured were excluded from the data analysis. Genotyping was carried out by laboratory staff blind to the MHT score results.

### Statistics

Medians are presented with inter-quartile ranges (IQR). The distributions of MHT scores by cohort are presented in kernel density plots. The Moray House Test score percentiles were normalised by transformation to an IQ type scale of mean 100 and standard deviation 15 using the invnorm function in STATA. This transformation facilitates the use of parametric tests and allows the magnitude of any genetic effect to be presented on a commonly understood scale in the field of cognition. Statistical analysis was carried out using STATA/SE version 11 (Stata Corp, College Station, Texas, USA) for both additive (CC vs CT vs TT) and dominant (CC vs CT/TT) models. Associations between genotype and intelligence were evaluated using various statistical tests; linear regression, logistic regression, Chi^2^. Genotype-sex interactions were assessed by Hosmer and Lemeshow likelihood ratio test. Regression coefficients (un-standardised) are presented with 95% confidence intervals and p values.

## Results

**Figure 1:**
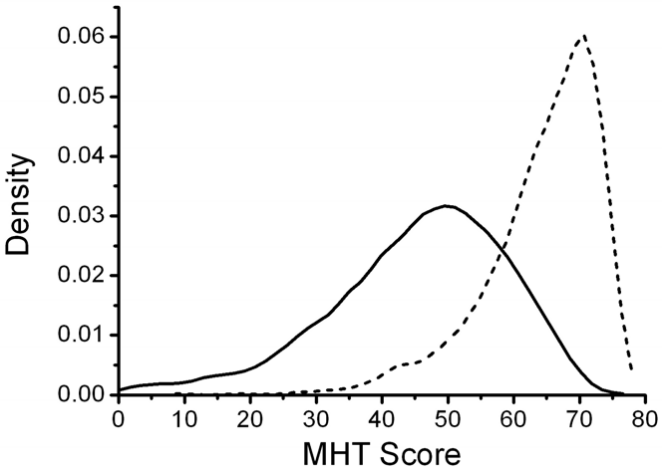
Kernel density plots of childhood Moray House test score in childhood (age 11yrs - - - -) and adulthood (age 70yrs ——) Moray House Test score.

The study population consisted of 50% males and 50% females. The median MHT score at age 11yrs in the entire sample was 47 (IQR = 17); 42 (IQR = 18) for ABC1936 and 50 (IQR = 15) for LBC1936. At age 70yrs the median MHT score was 66 (IQR = 11). The population distributions of childhood, and adult scores are shown in **Figure 1**. The scores were standardised to an IQ type scale (mean of 100, standard deviation of 15) for regression analysis; the levels of significance were similar after using other transformations to produce normally distributed MHT data (e.g. squaring in the case of childhood MHT score). The genotype and allele frequencies for the *DNMT* variants are shown in **Table 1**. All the variants studied were in Hardy-Weinberg equilibrium in the combined study population and the individual cohorts (tested by Chi^2^). The results of linear regression analysis of DNMT genotype on childhood and adult MHT scores are presented in **Table 2**. Of the four gene variants studied only *DNMT3L* was liked to intelligence. The minor allele was associated with a higher level of childhood intelligence in the additive model (coefficient  = 1.40; 95%CI 0.22,2.59; p = 0.020). The *DNMT3L* effect size was larger, and the level of significance greater, in the dominant model which compares carriers of the minor allele (CT/TT) with the homozygote CC (coefficient  = 1.99; 95%CI 0.55,3.43; p = 0.007). The *DNMT3L* genotype accounted for around 0.5% of the variance in intelligence, with the common homozygote (*DNMT3L* 11330CC) being associated with an approximately 2 point reduction in standardised intelligence. There was no evidence of genotype-sex interaction in relation to childhood intelligence score.

**Table 1:** *DNMT* variant genotype and allele frequencies in ABC1936 and LBC1936.

	Gene (variant)			
	*DNMT*(21220C>T)	*DNMT3A*(28510C>T)	*DNMT3B*(46359C>T)	*DNMT3L*(11330C>T)
Homozygote common	350 (22.8)	898 (58.3)	481 (31.2)	813 (53.8)
Heterozygote	795 (51.7)	544 (35.3)	753 (48.9)	607 (40.2)
Homozygote minor	393 (25.6)	99 (6.4)	307 (19.9)	92 (6.1)
Total	1538 (100)	1541 (100)	1541 (100)	1512 (100)
Common allele frequency	0.49	0.76	0.56	0.74
Minor allele frequency	0.51	0.24	0.44	0.26

Genotype counts are presented with percentages in brackets.

**Table 2:** Relationship between *DNMT* variants and standardised^1^ Moray House Test (MHT) score.

	Gene (variant)							
	*DNMT1*(21220C>T)		*DNMT3A*(28510C>T)		*DNMT3B*(46359C>T)		*DNMT3L*(11330C>T)	
Comparison	Regression coefficient(95% CI)	p value	Regression coefficient(95% CI)	p value	Regression coefficient(95% CI)	p value	Regression coefficient(95% CI)	p value
**Childhood intelligence**								
Additive model^2^	0·73(−0·30,1·76)	0·167	0·88(−0·28,2·04)	0·135	0·78(−0·24,1·80)	0·132	1·40(0·22,2·59)	0·020
Dominant model^3^	0·76(−0·95,2·46)	0·385	0·73(−0·72,2·17)	0·325	−1.31(−0·24,2·86)	0·097	1·99(0·55,3·43)	0·007
**Adult intelligence**								
Additive model^2^	−0.11(−1·40,1·17)	0·863	1.11(−0·33,2·54)	0·130	−0·03(−1·30,1·25)	0·969	1·60(0·14,3·05)	0·032
Dominant model^3^	0·10(−2·07,2·26)	0·929	0·89(−0·91,2·69)	0·330	0·21(−1·73,2·15)	0·830	2·25(0·46,4·05)	0·014

1 Transformed to normalised IQ type scale of mean 100 and standard deviation 15.

2 TT>CT>CC.

3 CT/TT>CC.

**Figure 2:**
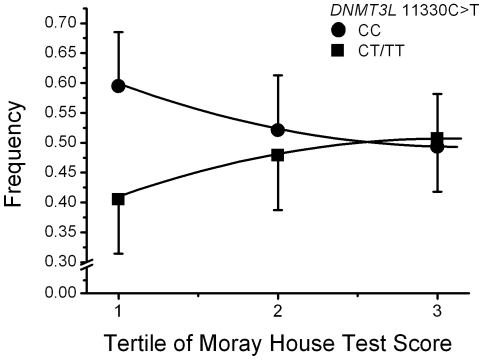
*DNMT3L* 11330C>T genotype frequencies – CC and T allele carriers (CT/TT) – by MHT score tertile.

The change in genotype frequency with tertile of intelligence is illustrated in **Figure 2** for the dominant model (p  =  0.005 by chi^2^). The *DNMT3L* change in CC frequency with intelligence was more pronounced at lower levels of intelligence. Logistic regression analysis of the effect of the CC variant on the likelihood of falling in the lowest intelligence group is presented for a range of centiles in Table 3. The *DNMT3L* 11330 C allele was associated with a higher risk of being below average intelligence – the lowest 50^th^ centile – in both the additive (OR 1.25; 95%CI 1.05,1.51; p = 0.011) and dominant (OR 1.37; 95%CI 1.11,1.68; p = 0.003) models. The effects sizes and levels of significance were similar when considering the lowest 40^th^ centile (OR 1.27_additive_; 95%CI 1.06,1.51; p = 0.009 and OR 1.40_dominant_; 95%CI 1.23,1.73; p = 0.002) or the lowest 30^th^ centile (OR 1.34_additive_; 95%CI 1.10,1.63; p = 0.004 and OR 1.44_dominant_; 95%CI 1.14,1.82; p = 0.002). For the 20^th^ centile the odds ratios were similarly positive but with one of the comparison groups consisting of only 20% of the data the number of observations was too small to demonstrate significance.

**Table 3:** *DNMT3L* 11330 genotype and risk of being in the lowest Moray House Test score centile.

	Additive model^1^		Dominant model^2^	
Lowest centile	Odds ratio(95% CI)	p value	Odds Ratio(95% CI)	p value
50^th^ centile	1.25(1.05,1.51)	0.011	1.37(1.11,1.68)	0.003
40^th^ centile	1.27(1.06,1.51)	0.009	1.40(1.23,1.73)	0.002
30^th^ centile	1.34(1.10,1.63)	0.004	1.44(1.14,1.82)	0.002
20^th^ centile	1.20(0.96,1.51)	0.114	1.23(0.93,1.62)	0.140

1 *DNMT3L* 11330 CC>CT>TT.

2 *DNMT3L* 11330 CC>CT/TT.

Adult intelligence was also related to *DNMT3L* genotypes (**Table 2**). The minor allele was associated with a higher level of adult intelligence in both the additive (coefficient 1.60; 95%CI 0.14,3.05; p = 0.032) and dominant (coefficient 2.25; 95%CI 0.46,4.05; p = 0.014) models. There was no evidence of genotype-sex interaction in relation to adult intelligence score. Although the level of significance was lower than for the childhood association with *DNMT3L* genotype it should be noted that this analysis was based only on LBC1936 therefore the numbers were significantly reduced. Interpretation of the adult data is also complicated by the fact that around 95% of the adults improved on their childhood intelligence score with a median improvement of 15 (IQR = 10) score points. This means that the most able children are likely to be limited by the nature of the test in adult life, resulting in a reduction in the power of the test to discriminate intelligence at the highest levels (ceiling effect) and a skewed distribution of scores (**Figure 1**). There is no way of knowing from the distribution alone which adults would be affected in this way therefore no single cut-off value can be derived. However, we can say that the relationship between adult intelligence and DNMT3L genotype remained significant, by both the additive and dominant models, following sequential exclusion of the highest MHT scores over a large span of the data; from the maximum possible score of 76 down to a score of 67 using the additive model and down to a score of 59 using the dominant model. At lower cut-off values the amount of data on which the analysis was based was too low to provide a meaningful test.

We tested here for the influence of 4 independent gene variants on intelligence. After Bonferroni correction for the number variants tested the minor T allele remained associated with a higher level of childhood intelligence (homozygote CC associated with a lower level of intelligence) by linear regression (p = 0.028). The chi^2^ analysis of the change in minor allele frequency with childhood intelligence tertile remained significant (p for trend  =  0.020). The risk of being in the lowest intelligence centile associated conferred by the CC genotype also remained significant across the same range of values for both the additive and dominant models; below average intelligence (p = 0.044_additive_, p = 0.012_dominant_);lowest 40^th^ centile (p = 0.036_additive_, p = 0.008_dominant_); lowest 30^th^ centile (p = 0.016_additive_, p = 0.008_dominant_). After Bonferroni correction the minor T allele association with adult intelligence by linear regression was only approaching significance (p = 0.056).

## Discussion

We observed a significant association between the *DNMT3L* 11330C>T variant and childhood intelligence in a study population made up of two large birth cohorts born in Scotland in 1936. Adult intelligence was also related to the same *DNMT3L* genotype but it should be noted that the test of intelligence, designed for children, may not have been sufficiently challenging to discriminate adult performance at the highest levels of intelligence and the number of data points on which the analysis was based (LBC1936 only) was less than for the childhood data. The relationship between DNMT3L and adult intelligence was only approaching statistical significance after Bonferroni adjustment therefore the primary finding here is in relation to childhood intelligence.

Intelligence is a highly complex phenotype which is the net result of a wide rage of biological processes and the effect of individual polymorphisms on intelligence is thought to be very low [3]. In this study the *DNMT3L* genotype accounted for around 0.5% of the variance in intelligence, with the common DNMT3L homozygote genotype being associated with an approximately 2 point reduction in standardised intelligence. It should be emphasised that this variant could not be used as any sort of test or predictor of intelligence, at either the group or individual level. The value of this finding is that it may point to specific biological processes which are worthy of further study. Current understanding of the role of DNMT3L points to the process of epigenetic regulation and imprinting – parent of origin specific epigenetic marking – in particular [21], [29], [30]. DNMT3L is known to interact with the histone deacetylases [31] and histone methyltransferases [32] but its primary effect is on *de novo* DNA methylation [21], [29], [30]. DNMT3L is structurally similar to the other methyltransferases. It is essential for de novo methylation but its mode of action primarily involves interaction with the other methyltransferases as it does not have methyltransferase activity itself [18]–[22]. Studies in animals have demonstrated that the progeny of *Dnmt3L* knockouts exhibit loss of imprinting, stochastic imprinting and biallelic expression of imprinted genes [33], [34]. The *DNMT3L* 11330C>T variant alters the amino acids sequence of the C-terminal portion of DNMT3L which interacts with the active catalytic methyltransferase domain of DNMT3A and DNMT3B [19]. A recent study reported differences in the methylation level of some genes in association with the *DNMT3L* 11330C>T variant [26]. These differences were not significant after adjustment for multiple testing but it is unlikely that this study was sufficiently powered for the very large number of tests carried out. The imprint is set during reproduction and, in a human study analogous to the experimental animal knockouts, we measured the effect of maternal DNMT3L 11330C>T genotype on the methylation status of the imprinted gene IGF2 in newborn cord blood (manuscript in preparation). The minor DNMT3L allele, associated here with a higher level of adult intelligence, was associated with a significantly lower level of methylation in IGF2 using the same statistical model (p = 0.035, n = 840).

A mechanism operating through epigenetic regulation, and imprinting in particular, would be consistent with many of the characteristics of human intelligence. Although known imprinted genes make up only around 1% of all genes in humans they primarily affect brain function and behaviour and pre-natal growth [5], [35]. These two key effects of the imprinted genes are consistent with the epidemiological link between IQ and birth weight [36]. The differential maternal and paternal inheritance patterns of many mental disorders would also be consistent with an imprinting mechanism [37], [38]. Direct evidence of an imprinting link to intelligence comes from disorders of imprinting, such as Prader-Willi syndrome (MIM176270) and Angelman syndrome (MIM105830), which are associated with a reduction in IQ [12]. Other mental impairment syndromes have been linked to imprinting changes or genetic polymorphisms relevant to epigenetics [9], [11], [13], [39], [40]. Evidence in support of a role for imprinting in neural function and cognition continues to grow [5], [41]–[47] but direct evidence in humans is difficult to obtain because of the difficulty of studying the brain directly. A link between intelligence and a genetic variant within a gene which is critical to imprinting is therefore a useful piece of additional evidence.

The potential involvement of epigenetics, and imprinting in particular, raises the intriguing possibility that even the heritable component of intelligence could be modifiable by factors such as diet during early development. The ultimate methyl donor for epigenetic-methylation reactions is the folate-methylation cycle and feeding pregnant dams diets deficient in methyl donors results in altered epigenetic regulation of specific genes in the offspring; e.g. axin fused [48] and the Agouti gene which is under imprinting control [49], [50]. Variation in the expression and epigenetic marking of the imprinted genes is also seen in humans [35], [51], [52] and human twin studies have demonstrated the heritability of imprinted gene methylation [53], [54]. We speculate that these two properties of imprinting – heritability and plasticity – could potentially explain the apparent paradox of the high level of IQ heritability [2] alongside the steady rise in IQ test scores from one generation to the next in the so called “Flynn effect” [55], [56].

The association between genetic variation in *DNMT3L* and childhood intelligence reported here must be considered an initial and as yet un-replicated finding, with the priority being to replicate the association in other populations. An association between *DNMT3L* and intelligence would be consistent with the critical role of *DNMT3L* in imprinting and the evidence linking imprinting to cognitive function but more work is needed to determine which function of DNMT3L is influenced by the 11330C>T variant and to investigate how this process might influence human intelligence.
